# Lymphocytes influence intracranial aneurysm formation and rupture: role of extracellular matrix remodeling and phenotypic modulation of vascular smooth muscle cells

**DOI:** 10.1186/s12974-016-0654-z

**Published:** 2016-07-14

**Authors:** David M. Sawyer, Lauren A. Pace, Crissey L. Pascale, Alexander C. Kutchin, Brannan E. O’Neill, Robert M. Starke, Aaron S. Dumont

**Affiliations:** Department of Neurosurgery, Tulane Center for Clinical Neurosciences, Tulane University School of Medicine, 131 S. Robertson St. Ste. 1300, 8047, New Orleans, LA 70112 USA; Department of Neurosurgery, University of Miami, Miami, FL USA

**Keywords:** Leukocyte, Interleukin-6, Inflammation, Cerebral aneurysm, Subarachnoid hemorrhage, Matrix metalloproteinase 2, Matrix metalloproteinase 9, Myosin heavy chain, Rag1 mice

## Abstract

**Background:**

Intracranial aneurysms (IA) are increasingly recognized as a disease driven by chronic inflammation. Recent research has identified key mediators and processes underlying IA pathogenesis, but mechanistic understanding remains incomplete. Lymphocytic infiltrates have been demonstrated in patient IA tissue specimens and have also been shown to play an important role in abdominal aortic aneurysms (AAA) and related diseases such as atherosclerosis. However, no study has systematically examined the contribution of lymphocytes in a model of IA.

**Methods:**

Lymphocyte-deficient (Rag1) and wild-type (WT; C57BL/6 strain) mice were subjected to a robust IA induction protocol. Rates of IA formation and rupture were measured, and cerebral artery tissue was collected and utilized for histology and gene expression analysis.

**Results:**

At 2 weeks, the Rag1 group had significantly fewer IA formations and ruptures than the WT group. Histological analysis of unruptured IA tissue showed robust B and T lymphocyte infiltration in the WT group, while there were no differences in macrophage infiltration, IA diameter, and wall thickness. Significant differences in interleukin-6 (IL-6), matrix metalloproteinases 2 (MMP2) and 9 (MMP9), and smooth muscle myosin heavy chain (MHC) were observed between the groups.

**Conclusions:**

Lymphocytes are key contributors to IA pathogenesis and provide a novel target for the prevention of IA progression and rupture in patients.

## Background

Intracranial aneurysms (IA) are important cerebrovascular diseases with a prevalence of 1–5 % [1, 2] although the incidence increases to 19 % in high-risk populations [3]. Approximately 6–20 per 100,000 patients per year will experience a life-threatening subarachnoid hemorrhage (SAH) as the result of IA rupture [4–8]. Although SAH accounts for only 3–8 % of all strokes [9], it is a disproportionate contributor to stroke burden. The prognosis of SAH patients is poor with a fatality rate of up to 50 % making it a significant contributor to stroke deaths in a relatively young population with a mean age of 50 years [10–12]. There is a critical need for better understanding of IA pathogenesis in order to provide improved early diagnosis and pharmacological treatment alternatives to patients who are at high risk for surgical interventions.

Ongoing work to elucidate the mechanisms behind IA pathogenesis promises to provide new targets for the diagnosis and treatment of this disease. Current understanding characterizes IA as a chronic inflammatory disease. The initiation of pathogenesis likely occurs with damage to the endothelium of the blood vessels in the cerebral circulation which recruits an inflammatory response that ultimately compromises the integrity of the vessel wall [13]. Key features of this process include a loss of the internal elastic lamina, myointimal hyperplasia, and ectopic distribution of vascular smooth muscle cells (VSMC) [14]. Many of these changes are influenced by the infiltration of leukocytes into the vessel wall which can act to degrade the extracellular matrix (ECM) through the secretion of MMPs and promote VSMC phenotypic modulation via secretion of cytokines such as tissue necrosis factor (TNF), interleukin-1 beta, and interleukin-6 (IL-6) [13]. Activated VSMC undergo dedifferentiation which is characterized by a reduction in contractile and cytoskeletal gene expression and increased expression of genes involved in the proliferation, migration, and matrix remodeling [15]. Inflammatory cell infiltration (macrophages and T lymphocytes and subsequently B lymphocytes) is followed by tissue fibrosis which is present in all ruptured IA [16].

The role of macrophages in IA formation and rupture has been well-characterized [17], yet the presence of other leukocyte populations has been noted in both IA and abdominal aortic aneurysms (AAA). A study of patient AAA tissue showed a reduction of VSMC markers, macrophage infiltration, and increased CD4+ and CD8+ T lymphocyte populations compared to healthy aorta. The presence of these inflammatory cells was also correlated with high levels of DNA fragmentation in VSMC [18]. There is also evidence that lymphocytes are localized in IA lesions suggesting that they may play a role in the inflammatory cascade underlying the disease. Studies of human tissue resected during clipping of IAs have shown that both T and B lymphocytes robustly infiltrate the vessel wall, preferentially around areas of IA rupture [19, 20]. Additionally, the presence of T lymphocytic infiltrate is associated with areas of myointimal hyperplasia in ruptured IA [21]. Although clear relationships have been demonstrated, it is impossible to determine from these studies whether lymphocytes exert a causal effect on the formation or rupture of IA.

Lymphocytes have been shown to have important effects in aneurysm-related inflammatory diseases such as atherosclerosis. T lymphocytes promote inflammation in atherosclerotic plaques via secretion of TNF and interferon gamma (IFNγ) which activates macrophages and B lymphocytes and increases expression of endothelial adhesion molecules [22–24]. B lymphocytes are involved in modulating the uptake of oxidized lipids by macrophages and can also contribute to endothelial damage [14]. Atherosclerotic lesions are found in all cerebral aneurysms histologically, and progression of atherosclerosis has correlated positively with aneurysm growth [20]. However, the mechanisms by which lymphocytes may contribute to cerebral aneurysm pathogenesis remain undefined.

The aim of the present study was to examine the role of lymphocytes in IA pathogenesis using a well-characterized murine model of IA. The experiments compared IA formation and rupture in mice deficient in lymphocytes (Rag1 knockouts, which do not produce mature B or T cells) to wild-type (WT) C57BL/6 mice. The tissue was collected from the animals for histology and gene expression analysis in order to examine the underlying mechanisms by which lymphocytes influence IA disease progression.

## Methods

### Animal model

Animal surgery procedures were approved by the Tulane Institutional Animal Care and Use Committee. Eight-week-old WT and lymphocyte-deficient mice (Rag1, on C57BL/6 background) (Jackson Laboratories) were used to induce IA via a previously described method involving induced hypertension and stereotactic elastase injection [25, 26]. In brief, animals underwent a unilateral nephrectomy followed 1 week later by a single stereotactic injection of 35 mU of elastase (Sigma-Aldrich) into the cerebrospinal fluid of the basal cistern. A subcutaneous pellet of deoxycorticosterone acetate (50 mg, 21-day release, Innovative Research of America) was also placed during this second surgery, after which the animals were given water containing 1.0 % NaCl until the conclusion of the study. All survival surgeries were performed under anesthesia using 100 mg/kg ketamine/10 mg/kg xylazine.

Animals were monitored daily for changes in weight or neurological function and those that lost more than 2.0 g of body mass in a 24-h period or displayed significant neurologic impairment were euthanized and necropsied to confirm the occurrence of SAH. All remaining mice were euthanized 14 days after elastase injection. These animals were perfused via intracardiac injection with phosphate-buffered saline (PBS) followed by a 2 mg/ml bromophenol blue (Thermo Fisher Scientific) in 4.0 % gelatin (Sigma-Aldrich) solution which allowed for visualization of the cerebral vessels. Brain samples were collected, and the cerebral vessels were carefully evaluated by a blinded observer for the presence of IA and SAH [25] then digitally imaged and dissected using an Olympus SZX7 stereomicroscope. Aneurysms were defined as a localized outward bulging of the vascular wall whose diameter was greater than 1.5 times the parent artery diameter by two independent observers blinded to the animal cohort. The number and location of IA and ruptures were recorded, and the IA tissue was collected and stored at −80 °C.

### Flow cytometry

At the experimental endpoint, the spleen tissue from each animal was collected and homogenized in order to quantify the lymphocyte population in the WT group and confirm the lymphocyte-deficient phenotype of the Rag1 animals. The number of cells collected from each spleen was determined using a hemocytometer, after which cells were stained using fluorescein (FITC)- and phycoerythrin (PE)-conjugated antibodies to CD2 (T cells, FITC; BD Biosciences) and CD19 (B cells, PE; BD Biosciences) and analyzed using a Beckman-Coulter Gallios™ flow cytometer. An absolute number of lymphocytes collected from each animal was calculated using spleen cellularity and proportion of cells expressing lymphocyte markers.

### Histology

The IA tissue was embedded in O.C.T. (Tissue-Tek) and flash frozen in isopentane (2-methyl butane) pre-chilled to −176 °C in liquid nitrogen. Eight-micrometer cryosections were collected using a CM1860 cryostat (Leica Microsystems). For immunofluorescence, slides were post-fixed in acetone for 10 min at −20 °C then equilibrated to room temperature. All slides were stained using Tris-buffered saline/Tween 20 (TBS/T) solutions. Slides were rinsed with TBS/T, blocked in 5 % serum (species of the secondary antibody) for 30 min, stained with an anti-CD68 (1:100; Abcam) antibody, or double labeled with anti-CD3 (SP7, 1:100; Abcam) and anti-CD19 (6D5, 1:100; Abcam) antibodies for 2 h then rinsed in TBS/T. Alexa Fluor 488 rabbit anti-rat and 594 goat anti-rabbit (both 1:300; Life Technologies) secondary antibodies were used to label the tissue for 1 h followed by nuclear labeling with DAPI (1:300; Life Technologies) for 5 min. ProLong Gold mounting media (Life Technologies) was used to prevent photobleaching during imaging. Cryosections were also stained with a Verhoeff van Gieson (VVG) kit (Polysciences, Inc.) to label connective tissues. Images (×100 and ×200) were collected using an Olympus IX73 inverted microscope with identical capture settings for each stain.

Histomorphometry was performed on the CD68 and VVG stains using ImageJ software (National Institutes of Health). For the CD68 stain, three ×200 images for each animal were obtained at identical exposure times and analyzed by calculation of corrected tissue fluorescence (CTF) [27]. The area of the tissue section was traced and measured in square micrometers, and the integrated density, i.e., total fluorescent intensity, was determined for each traced region. Integrated density for unstained background was also measured. The CTF was calculated using the following formula:

$$ \mathrm{C}\mathrm{T}\mathrm{F} = \mathrm{Integrated}\ \mathrm{density} - \left(\mathrm{Area}\ \mathrm{of}\ \mathrm{selected}\ \mathrm{tissue} \times \mathrm{Mean}\ \mathrm{fluorescence}\ \mathrm{of}\ \mathrm{background}\ \mathrm{readings}\right) $$

For the VVG stain, IA circumference, inner and outer diameter, and wall thickness were measured in micrometers at the widest point for each animal using ×100 images. Averages were determined using three images per animal per group.

### Quantitative real-time RT-PCR

The circle of Willis tissue was collected and stored at −80 °C in RNAlater solution (Life Technologies). Samples were pooled for the treatment groups, homogenized using a Scilogex D160, and RNA was extracted with Trizol reagent (Life Technologies) according to the manufacturer’s instructions. DNase post-treatment was performed on the samples using a DNA-free™ kit (Life Technologies) to remove genomic DNA contamination. cDNA was prepared with 0.5 μg total RNA per reaction using a Transcriptor First Strand cDNA kit (Roche Diagnostics), and mRNA expression was quantified with SYBR Green (Roche Diagnostics) on a Light Cycler 96 real-time system (Roche Diagnostics) and normalized to beta actin gene expression. Fold change is expressed over WT native tissue. Experiments were performed in triplicates with *n* = 3 reactions per group. Primer sequences are listed in Table 1.

**Table 1:** Quantitative real-time RT-PCR primer sequences

Gene	Forward	Reverse
Beta actin	GATCATTGCTCCTCCTGAG	ATCGTACTCCTGCTTGCT
Tissue necrosis factor	TCGTAGCAAACCACCAAG	AGCCTTGTCCCTTGAAGA
Interferon-γ	GTATTGCCAAGTTTGAGGTC	AATCAGCAGCGACTCCTT
Interleukin-6	ATTCATATCTTCAACCAAGAGG	TCCTTAGCCACTCCTTCT
Matrix metalloproteinase 2	GGAGACAAGTTCTGGAGATA	GGTTATCAGGGATGGCATT
Matrix metalloproteinase 9	GACATCTTCCAGTACCAAG	CCACCTTGTTCACCTCAT
Smooth muscle myosin heavy chain	CAGCTTGTCAGGAAGGAATA	TGACAGCACCTTCTACCT
Smooth muscle actin	CTTTCATTGGGATGGAGTCA	GGCTGTGATCTCCTTCTG
Transgelin (SM22)	TGTTCCAGACTGTTGACCT	AGTTGGCTGTCTGTGAAGT

### Statistics

Rates of IA formation and rupture in each group were evaluated using a Fisher’s exact test while histomorphometry and RT-PCR data were analyzed with a Student’s *t* test or one-way ANOVA with Bonferroni post hoc test using SigmaPlot 11.2 software (Systat Software Inc.). All data are expressed as mean ± SEM, and *p* ≤ 0.05 is considered statistically significant.

## Results

### Lymphocyte-deficient animals develop fewer cerebral aneurysms and have a lower incidence of aneurysm rupture

**Fig. 1:**
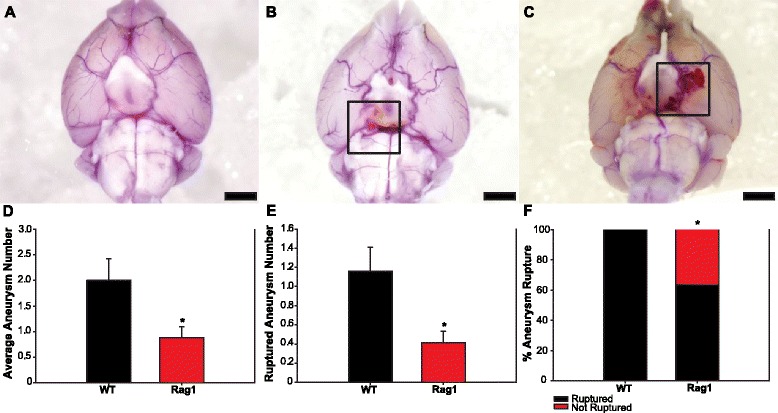
Rag1 knockout animals show less IA formation and rupture. Representative images of cerebral arteries at 14 days and quantification of IA formation and rupture. Scale bars = 2 mm. **a** No IA formation. **b** Unruptured IA. **c** Ruptured IA producing SAH. Rag1 group has significantly lower total IAs per animal (**d**) and ruptured IAs per animal (**e**). **f** Rag1 group has significantly lower rate of IA rupture. * *p* ≤ 0.05

Rates of IA formation and rupture were measured over the course of the experiment, and digital images of each brain were collected showing outcomes with no IA formation (Fig. 1a), IA formation (Fig. 1b), and IA rupture with SAH (Fig. 1c). The mean number of IAs formed per animal was 2.0 ± 0.42 in the WT group (*n* = 19) and 0.88 ± 0.21 in the Rag1 group (*n* = 17, *p* = 0.036) (Fig. 1d). The mean number of ruptures that occurred per animal was 1.16 ± 0.25 in the WT group (*n* = 19) and 0.41 ± 0.12 in the Rag1 group (*n* = 17, *p* = 0.026) (Fig. 1e). The rupture rate (percentage of animals that formed IAs in each group that suffered at least one rupture) was 100 % in the WT group (*n* = 16) and 63.6 % in the Rag1 group (*n* = 11, *p* = 0.031) (Fig. 1f).

### Rag1 animals exhibit similar macrophage infiltration but absent lymphocyte infiltration in cerebral aneurysms

**Fig. 2:**
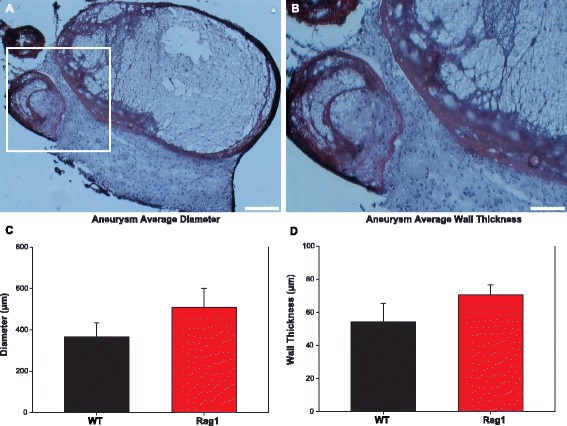
VVG stain for elastic fibers and quantification of IA diameter and wall thickness. Elastic fibers and nuclei (*black*) and collagen (*red*). **a** ×100 VVG and **b** ×200 representative image of Rag1 unruptured IA. Scale bars = 100 and 50 μm. No significant difference between average IA diameter (**c**) and wall thickness (**d**) between Rag1 and WT groups

**Fig. 3:**
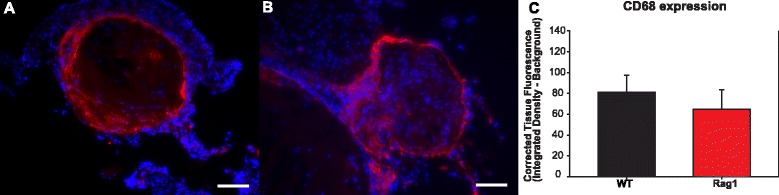
Macrophage CD68 immunostaining and quantification. **a** ×200 images of WT and **b** Rag1 IA tissue. Scale bars = 50 μm. **c** No significant differences in CD68 macrophage fluorescence between WT and Rag1 groups

The VVG stain allowed visualization of internal elastic lamina degeneration, thrombosis, and cellular infiltration for each group [28] (Fig. 2a, b). Quantification of unruptured IA diameter (Rag1 508.11 ± 92.10 μm, WT 367.14 ± 65.36 μm) and IA wall thickness (Rag1 70.60 ± 6.09 μm, WT 54.35 ± 11.08 μm) was not significantly different between the groups (Fig. 2c, d). Macrophage infiltration in the IA tissue was confirmed in all animals with the CD68 stain (Fig. 3a, b). CD68 CTF quantification for the WT group (81.21 ± 16.33 AU, *n* = 5) showed no significant differences from the Rag1 group (64.85 ± 18.60 AU, *n* = 6, *p* = 0.34, Fig. 3c).

**Fig. 4:**
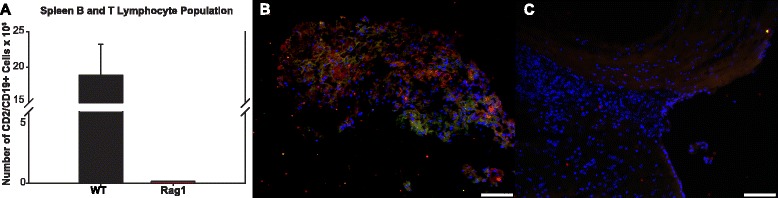
Splenic B and T lymphocyte populations and lymphocyte infiltration in WT IA tissue. **a** Number of CD19/CD2-positive B and T lymphocytes 2 weeks after IA induction. ×200 images of CD19 (*green*) and CD3 (*red*) double immunostaining show B and T lymphocyte infiltration in the WT (**b**) and not the Rag1 IA tissue (**c**). Scale bars = 50 μm

The lymphocyte-deficient phenotype of the Rag1 animals was confirmed by flow cytometry studies of spleen cell suspensions at the conclusion of the experiment. The proportion of spleen cells expressing lymphocyte markers (CD2 or CD19) was found to be 0.43 in the WT group (*n* = 9) and 0.043 in the Rag1 group (*n* = 10, *p* < 0.0001, data not shown). The mean number of cells expressing lymphocyte markers (lymphocyte proportion multiplied by spleen cellularity) was 18.88 × 10^6^ in the WT group and 0.14 × 10^6^ in the Rag1 group (Fig. 4a, *p* = 0.0003).

Robust B and T lymphocyte infiltration was confirmed in the WT group by immunofluorescent staining with CD19 and CD3 antibodies in the harvested IA tissue at 2 weeks (Fig. 4b) while no lymphocytes were present in the Rag1 tissue (Fig. 4c).

### Lymphocyte depletion alters vessel wall changes associated with cerebral aneurysm pathogenesis

**Fig. 5:**
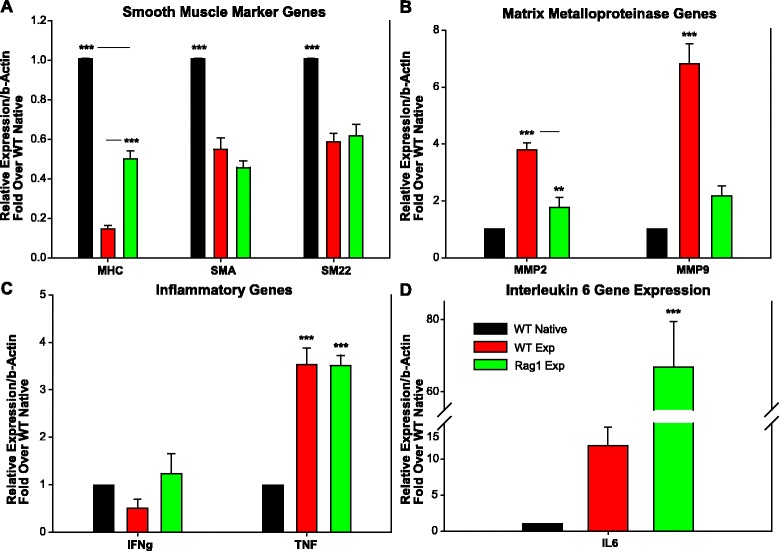
RT-PCR relative quantification of mRNA expression shows significant differences between the WT and Rag1 experimental groups. Smooth muscle marker genes (**a**), matrix metalloproteinases (**b**), and inflammatory genes (**c**, **d**). ** *p* ≤ 0.01; *** *p* ≤ 0.001

Quantitative RT-PCR mRNA expression was normalized to native uninjured cerebral artery tissue from WT animals (C57BL/6 native). Expression of VSMC marker genes smooth muscle alpha actin (SMA) and SM22 were significantly decreased in both animal groups (SMA fold change 0.55 ± 0.059 WT versus 0.46 ± 0.033 Rag1, SM22 fold change 0.59 ± 0.043 WT versus 0.62 ± 0.060 Rag1) compared to native tissue while MHC gene repression was attenuated in the Rag1 group (fold change 0.51 ± 0.037 versus 0.15 ± 0.015 WT, Fig. 5a).

IA induction increased the expression of matrix remodeling genes MMP2 and MMP9 in the WT group while this increase was significantly diminished in the Rag1 group (fold change 3.79 ± 0.25 MMP2, 6.82 ± 0.71 MMP9, *p* < 0.001, versus 2.23 ± 0.34 MMP2, 2.13 ± 0.33 MMP9 in the WT versus Rag1 group, respectively Fig. 5b).

IA induction did not alter IFNγ while TNFα was upregulated similarly in both groups (fold change 3.53 ± 0.35 and 3.51 ± 0.22, *p* < 0.001, versus WT native in the WT experimental and Rag1 experimental groups, respectively, Fig. 5c). IL-6 gene expression was significantly increased in the Rag1 group (fold change 66.83 ± 12.70, *p* < 0.001) compared to the WT experimental group (11.82 ± 2.61, Fig. 5d).

## Discussion

Lymphocytes are a well-defined component of chronic inflammation in many disease states and have been implicated as a contributing factor in vascular pathology such as atherosclerosis and AAA [14, 18, 29]. Although multiple investigators have reported the presence of these cells in patient IA tissue specimens, it was not previously known whether they play a causative role in the pathogenesis of the disease. This study used a well-characterized mouse model of IA to examine the effect of lymphocyte depletion on IA disease progression and its underlying mechanisms.

The present results provide evidence that lymphocytes are directly involved in the pathogenesis and rupture of IA. The Rag1 animals were confirmed to have negligible numbers of cells expressing lymphocyte markers in the spleen and in the walls of intracranial vessels, as compared with WT controls (Fig. 4). When subjected to the IA induction protocol, these lymphocyte-deficient animals displayed significantly reduced susceptibility to the disease. Rag1 animals formed fewer IAs than controls, suffered fewer ruptures, and demonstrated a lower rupture rate (Fig. 1). Histologically, the trend toward increased aneurysm size and wall thickness in the Rag1 group suggests that lymphocytes may be more involved in aneurysm rupture over formation. This will be explored in subsequent studies. It is likely that the significant differences in IA formation and rupture seen between the two groups may be the result of an altered inflammatory cell response in the Rag1 animals. The absence of lymphocytes did not completely halt the inflammatory process, but it may be possible that the lack of lymphocytic signaling molecules altered IA pathogenesis in this model. This process was investigated by gene expression analysis, which showed several key differences between the WT and Rag1 groups.

RT-PCR was performed on cerebral artery tissue collected from the groups at 2 weeks for VSMC marker, matrix remodeling, and inflammation genes. A histological study of saccular IAs in 66 patients found that increased VSMC proliferation and migration, decellularization, degeneration of ECM, and inflammatory cell infiltration were the distinctive factors between ruptured and unruptured tissue samples [21]. Animals that succumbed to SAH before the end of study were not included for histology and gene expression, in order to ensure a uniform endpoint of 2 weeks and because the possible delay between death and tissue collection would have resulted in degradation of the tissue. Separate analysis of ruptured and unruptured IA was not performed. Gene expression analysis of IA tissue at 2 weeks showed some key differences which may indicate the factors contributing to increased IA formation and rupture in the WT experimental group. IL-6 expression was found to be markedly increased in the Rag1 group compared to controls. IL-6 can act as a pro-inflammatory or anti-inflammatory cytokine depending on the identity of the bound receptor. Signaling via the IL-6 receptor (IL6R) has been shown to have anti-inflammatory effects, although only certain cell types, including macrophages, express this receptor. IL-6 binding to a soluble IL6R (sIL6R) complex with ubiquitously expressed membrane-bound signaling protein gp130 can initiate pro-inflammatory signaling in cells that do not express IL6R [30]. Increased systemic and local tissue expression of IL-6 has been well-characterized in clinical studies of IA, and although some suggest that lower IL-6 levels are synonymous with lower rates of IA rupture [31, 32], other studies have shown that certain polymorphisms in the IL-6 gene can be protective against IA [33]. A meta-analysis of IA in a Southern Indian population showed that genetic variants in TNF and IFNγ but not IL-6 were associated with IA [34]. In this study, a significantly lower rate of IA rupture was seen with markedly higher IL-6 tissue expression at 2 weeks. This could potentially implicate the anti-inflammatory role of IL-6 in a system lacking B and T lymphocytes, but more work is needed to confirm or refute this possibility. Future experiments involving Rag1 knockouts treated with or without IL-6 inhibitors could be useful in further elucidating the role of this signaling in IA. Expression of MMPs 2 and 9, gelatinases critical for ECM remodeling, was significantly lower in the Rag1 knockout tissue compared to the WT experimental group. These results are consistent with studies showing that decreased MMP9 expression correlates with lower IA rupture rates in a mouse elastase model. The role of MMP2 is less well-characterized as it is constitutively expressed [35, 36], and a mouse study of MMP2 knockouts showed no difference in IA incidence compared to WT controls [25]. Elevated expression of MMPs 2 and 9 have also been confirmed in patient IA tissue [37, 38], and Jin et al. found higher MMPs 2 and 9 expression in ruptured versus unruptured IA [39].

VSMC phenotypic modulation, proliferation, and migration from the tunica media are features of IA pathology. The VSMC dedifferentiate to a pro-inflammatory, pro-matrix remodeling phenotype, which is characterized by reduced expression of contractile proteins such as SMA and MHC and actin-binding proteins such as SM22 [15]. In this study, the reduction in expression of MHC was attenuated in Rag1 mice, although SMA and SM22 expression was similar between Rag1 and WT mice. It has been noted that SMA and SM22 may be expressed in other cell types following vascular injury [40, 41] while MHC is exclusively expressed in cells of myogenic lineage and is considered a more specific marker of VSMC phenotype [42]. Sibon et al. showed a time-dependent decrease in MHC but not SMA gene expression over 6 months in a rat carotid ligation model of IA [43]. The absence of lymphocytes in the Rag1 tissue may have impeded the phenotypic modulation in the VSMC resulting in lower expression of MMPs 2 and 9 and higher expression of MHC.

The present study is characterized by a number of limitations that should be addressed in future work. Gene expression data for MMPs were not followed by measurements of MMP activity due to tissue quantity limitations. The choice to use Rag1 knockout mice was effective in allowing the study of aneurysm formation in the complete absence of lymphocytes, but it is possible that these knockout animals could display developmental abnormalities or compensatory mechanisms that could influence IA pathogenesis in unpredictable ways. Additionally, there is some evidence to suggest that Rag1 knockouts display a moderated increase in blood pressure with DOCA administration [44]. Blood pressure measurements were not obtained in the current study due to institutional quarantine procedures for these immune-deficient animals undergoing multiple surgeries. Future studies examining the temporal course and magnitude and blood pressure changes are underway using implanted telemetry devices. However, hypertension is induced in this model via a multi-pronged approach, and the present data suggest that lymphocytes may contribute to IA pathogenesis through mechanisms unrelated to hypertension.

Following this novel identification of lymphocytes as a contributing factor to IA rupture, it will be important to further elucidate the specific mechanisms by which these cells exert their effects. At this point, it is unknown which subpopulations of lymphocytes are responsible—it would be appropriate for further experiments to test the contributions of T cells versus B cells, helper T cells, and regulatory T cells. These subpopulations have varying and contradictory effects on the inflammatory cascade [14], and it is likely that the study of lymphocytes as a general category in this experiment simplifies the contributions of these cells to the disease process. In addition, the relevant signaling pathways need to be further characterized. Identification of these mechanistic underpinnings could provide the basis for the development of therapeutics useful in the prevention of IA progression to rupture.

## Conclusions

These data suggest that lymphocytes play an important role in IA pathogenesis in a well-characterized mouse model. The lack of these cells led to altered VSMC activation following IA induction. More specifically, vessel wall changes contributing to the pathogenesis of IA, including increased expression of pro-matrix remodeling genes and a reduction in expression of MHC, were attenuated in Rag1 animals lacking lymphocytes compared to wild-type controls. Consequently, lymphocyte-deficient Rag1 animals demonstrated a significantly lower rate of aneurysm formation and rupture. Future studies to delineate mechanisms by which lymphocytes contribute to IA pathogenesis may uncover novel putative therapeutic targets.

## Abbreviations

AAA, abdominal aortic aneurysm; ECM, extracellular matrix; IA, intracranial aneurysm; IFNγ, interferon gamma; IL-6, interleukin-6; IL6R, interleukin-6 receptor; MHC, smooth muscle myosin heavy chain; MMP2, matrix metalloproteinase 2; MMP9, matrix metalloproteinase 9; SAH, subarachnoid hemorrhage; TNF, tissue necrosis factor; VSMC, vascular smooth muscle cells

## References

[1] Rinkel GJ (2008). Natural history, epidemiology and screening of unruptured intracranial aneurysms. J Neuroradiol.

[2] Komotar RJ, Zacharia BE, Mocco J, Connolly ES (2008). Controversies in the surgical treatment of ruptured intracranial aneurysms: the First Annual J. Lawrence Pool Memorial Research Symposium—controversies in the management of cerebral aneurysms. Neurosurgery.

[3] Brown RD, Huston J, Hornung R, Foroud T, Kallmes DF, Kleindorfer D, Meissner I, Woo D, Sauerbeck L, Broderick J (2008). Screening for brain aneurysm in the Familial Intracranial Aneurysm study: frequency and predictors of lesion detection. J Neurosurg.

[4] Brisman JL, Song JK, Newell DW (2006). Cerebral aneurysms. N Engl J Med.

[5] van Gijn J, Kerr RS, Rinkel GJ (2007). Subarachnoid haemorrhage. Lancet.

[6] Kassell NF, Torner JC (1984). The International Cooperative Study on Timing of Aneurysm Surgery—an update. Stroke.

[7] Schievink WI (1997). Intracranial aneurysms. N Engl J Med.

[8] Ronkainen A, Miettinen H, Karkola K, Papinaho S, Vanninen R, Puranen M, Hernesniemi J (1998). Risk of harboring an unruptured intracranial aneurysm. Stroke.

[9] Sudlow CL, Warlow CP (1997). Comparable studies of the incidence of stroke and its pathological types: results from an international collaboration. International Stroke Incidence Collaboration. Stroke.

[10] Johnston SC, Selvin S, Gress DR (1998). The burden, trends, and demographics of mortality from subarachnoid hemorrhage. Neurology.

[11] Krishnamurthi RV, Moran AE, Forouzanfar MH, Bennett DA, Mensah GA, Lawes CM, Barker-Collo S, Connor M, Roth GA, Sacco R (2014). The global burden of hemorrhagic stroke: a summary of findings from the GBD 2010 study. Glob Heart.

[12] de Rooij NK, Linn FH, van der Plas JA, Algra A, Rinkel GJ (2007). Incidence of subarachnoid haemorrhage: a systematic review with emphasis on region, age, gender and time trends. J Neurol Neurosurg Psychiatry.

[13] Chalouhi N, Ali MS, Jabbour PM, Tjoumakaris SI, Gonzalez LF, Rosenwasser RH, Koch WJ, Dumont AS (2012). Biology of intracranial aneurysms: role of inflammation. J Cereb Blood Flow Metab.

[14] Sawyer DM, Amenta PS, Medel R, Dumont AS (2015). Inflammatory mediators in vascular disease: identifying promising targets for intracranial aneurysm research. Mediators Inflamm.

[15] Starke RM, Chalouhi N, Ding D, Raper DM, McKisic MS, Owens GK, Hasan DM, Medel R, Dumont AS (2014). Vascular smooth muscle cells in cerebral aneurysm pathogenesis. Transl Stroke Res.

[16] Tulamo R, Frosen J, Hernesniemi J, Niemela M (2010). Inflammatory changes in the aneurysm wall: a review. J Neurointerv Surg.

[17] Hasan D, Chalouhi N, Jabbour P, Hashimoto T (2012). Macrophage imbalance (M1 vs. M2) and upregulation of mast cells in wall of ruptured human cerebral aneurysms: preliminary results. J Neuroinflammation.

[18] Henderson EL, Geng YJ, Sukhova GK, Whittemore AD, Knox J, Libby P (1999). Death of smooth muscle cells and expression of mediators of apoptosis by T lymphocytes in human abdominal aortic aneurysms. Circulation.

[19] Chyatte D, Bruno G, Desai S, Todor DR (1999). Inflammation and intracranial aneurysms. Neurosurgery.

[20] Kosierkiewicz TA, Factor SM, Dickson DW (1994). Immunocytochemical studies of atherosclerotic lesions of cerebral berry aneurysms. J Neuropathol Exp Neurol.

[21] Frosen J, Piippo A, Paetau A, Kangasniemi M, Niemela M, Hernesniemi J, Jaaskelainen J (2004). Remodeling of saccular cerebral artery aneurysm wall is associated with rupture: histological analysis of 24 unruptured and 42 ruptured cases. Stroke.

[22] Tse K, Tse H, Sidney J, Sette A, Ley K (2013). T cells in atherosclerosis. Int Immunol.

[23] Lintermans LL, Stegeman CA, Heeringa P, Abdulahad WH (2014). T cells in vascular inflammatory diseases. Front Immunol.

[24] Ammirati E, Moroni F, Magnoni M, Camici PG (2015). The role of T and B cells in human atherosclerosis and atherothrombosis. Clin Exp Immunol.

[25] Nuki Y, Tsou TL, Kurihara C, Kanematsu M, Kanematsu Y, Hashimoto T (2009). Elastase-induced intracranial aneurysms in hypertensive mice. Hypertension.

[26] Starke RM, Chalouhi N, Jabbour PM, Tjoumakaris SI, Gonzalez LF, Rosenwasser RH, Wada K, Shimada K, Hasan DM, Greig NH (2014). Critical role of TNF-alpha in cerebral aneurysm formation and progression to rupture. J Neuroinflammation.

[27] Burgess A, Vigneron S, Brioudes E, Labbe JC, Lorca T, Castro A (2010). Loss of human Greatwall results in G2 arrest and multiple mitotic defects due to deregulation of the cyclin B-Cdc2/PP2A balance. Proc Natl Acad Sci U S A.

[28] Keedy A (2006). An overview of intracranial aneurysms. Mcgill J Med.

[29] Ocana E, Bohorquez JC, Perez-Requena J, Brieva JA, Rodriguez C (2003). Characterisation of T and B lymphocytes infiltrating abdominal aortic aneurysms. Atherosclerosis.

[30] Scheller J, Chalaris A, Schmidt-Arras D, Rose-John S (1813). The pro- and anti-inflammatory properties of the cytokine interleukin-6. Biochim Biophys Acta.

[31] Shimada K, Furukawa H, Wada K, Korai M, Wei Y, Tada Y, Kuwabara A, Shikata F, Kitazato KT, Nagahiro S (2015). Protective role of peroxisome proliferator-activated receptor-gamma in the development of intracranial aneurysm rupture. Stroke.

[32] Kao HW, Lee KW, Kuo CL, Huang CS, Tseng WM, Liu CS, Lin CP (2015). Interleukin-6 as a prognostic biomarker in ruptured intracranial aneurysms. PLoS One.

[33] McColgan P, Thant KZ, Sharma P (2010). The genetics of sporadic ruptured and unruptured intracranial aneurysms: a genetic meta-analysis of 8 genes and 13 polymorphisms in approximately 20,000 individuals. J Neurosurg.

[34] Sathyan S, Koshy LV, Srinivas L, Easwer HV, Premkumar S, Nair S, Bhattacharya RN, Alapatt JP, Banerjee M (2015). Pathogenesis of intracranial aneurysm is mediated by proinflammatory cytokine TNFA and IFNG and through stochastic regulation of IL10 and TGFB1 by comorbid factors. J Neuroinflammation.

[35] Pena Silva RA, Kung DK, Mitchell IJ, Alenina N, Bader M, Santos RA, Faraci FM, Heistad DD, Hasan DM (2014). Angiotensin 1-7 reduces mortality and rupture of intracranial aneurysms in mice. Hypertension.

[36] Wakisaka Y, Chu Y, Miller JD, Rosenberg GA, Heistad DD (2010). Spontaneous intracerebral hemorrhage during acute and chronic hypertension in mice. J Cereb Blood Flow Metab.

[37] Caird J, Napoli C, Taggart C, Farrell M, Bouchier-Hayes D (2006). Matrix metalloproteinases 2 and 9 in human atherosclerotic and non-atherosclerotic cerebral aneurysms. Eur J Neurol.

[38] Bruno G, Todor R, Lewis I, Chyatte D (1998). Vascular extracellular matrix remodeling in cerebral aneurysms. J Neurosurg.

[39] Jin D, Sheng J, Yang X, Gao B (2007). Matrix metalloproteinases and tissue inhibitors of metalloproteinases expression in human cerebral ruptured and unruptured aneurysm. Surg Neurol.

[40] Li L, Miano JM, Cserjesi P, Olson EN (1996). SM22 alpha, a marker of adult smooth muscle, is expressed in multiple myogenic lineages during embryogenesis. Circ Res.

[41] Caplice NM, Bunch TJ, Stalboerger PG, Wang S, Simper D, Miller DV, Russell SJ, Litzow MR, Edwards WD (2003). Smooth muscle cells in human coronary atherosclerosis can originate from cells administered at marrow transplantation. Proc Natl Acad Sci U S A.

[42] Miano JM, Cserjesi P, Ligon KL, Periasamy M, Olson EN (1994). Smooth muscle myosin heavy chain exclusively marks the smooth muscle lineage during mouse embryogenesis. Circ Res.

[43] Sibon I, Mercier N, Darret D, Lacolley P, Lamaziere JM (2008). Association between semicarbazide-sensitive amine oxidase, a regulator of the glucose transporter, and elastic lamellae thinning during experimental cerebral aneurysm development: laboratory investigation. J Neurosurg.

[44] Guzik TJ, Hoch NE, Brown KA, McCann LA, Rahman A, Dikalov S, Goronzy J, Weyand C, Harrison DG (2007). Role of the T cell in the genesis of angiotensin II induced hypertension and vascular dysfunction. J Exp Med.

